# Impairments of Motor Function While Multitasking in HIV

**DOI:** 10.3389/fnhum.2017.00212

**Published:** 2017-04-28

**Authors:** Sharif I. Kronemer, Jordan A. Mandel, Ned C. Sacktor, Cherie L. Marvel

**Affiliations:** ^1^Department of Neurology, Johns Hopkins School of MedicineBaltimore, MD, USA; ^2^Department of Neuroscience, Yale UniversityNew Haven, CT, USA; ^3^Department of Psychiatry and Behavioral Sciences, Johns Hopkins University School of MedicineBaltimore, MD, USA

**Keywords:** HIV, multitasking, motor, working memory, cognition, HIV-associated neurocognitive disorders (HAND)

## Abstract

Human immunodeficiency virus (HIV) became a treatable illness with the introduction of combination antiretroviral therapy (CART). As a result, patients with regular access to CART are expected to live decades with HIV. Long-term HIV infection presents unique challenges, including neurocognitive impairments defined by three major stages of HIV-associated neurocognitive disorders (HAND). The current investigation aimed to study cognitive and motor impairments in HIV using a novel multitasking paradigm. Unlike current standard measures of cognitive and motor performance in HIV, multitasking increases real-world validity by mimicking the dual motor and cognitive demands that are part of daily professional and personal settings (e.g., driving, typing and writing). Moreover, multitask assessments can unmask compensatory mechanisms, normally used under single task conditions, to maintain performance. This investigation revealed that HIV+ participants were impaired on the motor component of the multitask, while cognitive performance was spared. A patient-specific positive interaction between motor performance and working memory recall was driven by poor HIV+ multitaskers. Surprisingly, HAND stage did not correspond with multitask performance and a variety of commonly used assessments indicated normal motor function among HIV+ participants with poor motor performance during the experimental task. These results support the use of multitasks to reveal otherwise hidden impairment in chronic HIV by expanding the sensitivity of clinical assessments used to determine HAND stage. Future studies should examine the capability of multitasks to predict performance in personal, professional and health-related behaviors and prognosis of patients living with chronic HIV.

## Introduction

Human immunodeficiency virus (HIV) has killed 34 million people worldwide since the late 20th century (World Health Organization, 2015). In the United States 1.2 million people are currently diagnosed with HIV and an additional 50,000 people are newly infected each year (Centers for Disease Control and Prevention, 2014). The prognosis for people with HIV changed dramatically in the United States with the introduction of combination antiretroviral therapy (CART) in the mid-1990s (Palella et al., 1998). CARTs offered the possibility to chronically manage HIV and greatly increased longevity. The life expectancy for North Americans with HIV is approximately 71 years, near the life expectancy of the general population (Samji et al., 2013). Similar life expectancies are reported among people with HIV in other countries with reliable access to CART (May et al., 2014).

Given that HIV+ individuals are living longer due to CART, it is now the case that half of all HIV+ people in the United States are over 50 years old. Yet, the long-term impacts of HIV are only beginning to be explored (Greene et al., 2013). A common outcome of chronic HIV is neurocognitive dysfunctions, also referred to as HIV-associated neurocognitive disorders (HAND). Approximately 50 percent of HIV+ patients experience HAND (Heaton et al., 2010). While CARTs have decreased incidences of HIV-associated dementia (HAD), or AIDS dementia complex, there remains a high prevalence of patients who experience milder forms of HAND, including asymptomatic neurocognitive impairment (ANI) and minor neurocognitive disorder (MND; McArthur et al., 2010; Schouten et al., 2011).

The AIDS Task Force of the American Academy of Neurology (AAN) initially established the foundational criteria that define neurocognitive stages of HIV (Janssen et al., 1991). The AAN criteria were criticized in part for neglecting milder forms of impairment, for example how multiple impairments that individually do not breach a threshold of abnormality on clinical assessments can compound to become a notable functional deficit (Antinori et al., 2007). In response to these limitations, the original AAN criteria were revised in Frascati, Italy and the updated “Frascati” criteria defined the ANI category to capture patients with milder symptoms that may later progress to more severe stages of HAND (Antinori et al., 2007). The ANI category offers the opportunity for proactive treatments and behavioral modifications that are protective from advancements in HAND. Attention to milder forms of HAND and early detection of neurocognitive impairments should be a priority as data suggests an increased frequency of ANI among HIV+ gay and bisexual men in the US (Sacktor et al., 2016).

Of the cognitive functions impaired in HAND, working memory deficits are particularly salient. People with HIV exhibit decreased verbal and visual working memory capacity (Martin et al., 2001; York et al., 2001; Grant, 2008; Sun et al., 2010; Sundermann et al., 2015). Likewise, neuroimaging data reveals HIV preferentially targets cortical structures that drive memory, including the prefrontal cortex, basal ganglia and hippocampus (Aylward et al., 1995; Castelo et al., 2006; McNab and Klingberg, 2008; Hoare et al., 2012; Thames et al., 2012). These memory deficits are distinctly harmful in HIV because working memory is vital to maintain the goal-directed behaviors necessary for health with chronic HIV, including adherence to CART schedules (Insel et al., 2006) and inhibiting risky or impulsive behaviors (e.g., drug use or unprotected sex; Anderson et al., 2013, 2016) that can increase viral load, suppress the immune system, and lead to HIV transmission (Siddiqui et al., 1993; Roth et al., 2002).

In addition to cognitive impairments, motor abilities are recurrently compromised with chronic HIV. The most frequent motor disorder in HIV is Parkinsonism (Bhidayasiri and Tarsy, 2012). Milder motor deficits are also reported, including isolated eye movement impairment (Sweeney et al., 1991), reduced motor speed (bradykinesia; Karlsen et al., 1992; Ogunrin and Odiase, 2006), impaired facial expression (hypomimia), and tremor (Valcour et al., 2008). Motor impairments in HIV are linked to disease of the basal ganglia and cortical-subcortical networks that serve motor functions (DeVaughn et al., 2015). In addition, primary and supplementary motor areas are shown to be less active in HIV seropositive subjects during movement tasks (Wilson et al., 2013). Motor impairment in HIV introduces potential health risks, for example, falls and motor vehicle accidents (Marcotte et al., 1999; Erlandson et al., 2012), and may prevent the patient from performing personal or professional responsibilities.

As outlined above, people infected with HIV can exhibit cognitive and motor impairment. The assessments commonly used to evaluate neurocognitive impairment in HIV primarily examine function within distinct domains (Schouten et al., 2011). HAND scores and biomarkers of HIV (e.g., viral load, CD4 nadir, CART adherence, etc.) do not consistently predict performance on assessments of cognition and motor performance (Anderson et al., 2016). In addition, the AAN criteria and revisions by Antinori et al. (2007) are limited by a reliance upon single modality clinical assessments that may not capture impairments observed while the HIV+ patient is performing activities in daily life (Scott et al., 2011). This suggests that current neurocognitive assessments in HIV may require improvements to increase sensitivity to deficits that can be masked during routine clinic evaluations. In response, the current investigation examines impairments of cognitive and motor function in HIV through a multitasking, or dual task, paradigm. This approach offers greater ecological validity than most current neurocognitive assessments, as many daily activities will recruit both cognitive and motor systems simultaneously (e.g., cooking, driving, reading, typing, walking and writing). Limited research has been devoted to evaluate multitasking in HIV. Previous investigations have examined multitasking by combining several cognitive tasks and have reported deficits (e.g., Scott et al., 2011). However, combining cognitive with motor tasks may be a more accurate reflection of real world function because many activities require both domains at once. Indeed, patients with HIV complain of challenges with multitasking in professional and personal settings (Schouten et al., 2011). The results generated from a cognitive-motor multitask assessment may, therefore, be more sensitive to HIV-associated neurocognitive impairment than are the standard tests currently used in clinical assessments.

We developed a novel cognitive-motor multitask paradigm that engages fine motor and working memory functions simultaneously. We hypothesized that HIV+ participants would show reduced working memory capacity and decreased fine motor ability as a function of cognitive-motor dual load. Importantly, impairments would be measured on a more sensitive scale compared to that of standardized single modality assessments.

## Materials and Methods

### Participants

Twenty-five HIV+ participants (41–68 years, mean = 57.96 years, 6 females; see Table 1) were recruited from Johns Hopkins HIV Neurology Clinical Core Cohort (Gandhi et al., 2010). The patients had been diagnosed with HIV for an average of 20.6 years (6–36 years). All patients maintained a daily CART schedule. HAND stage, clinical data (e.g., CD4 level and CART regiment), and neuro-medical exams of motor function, including the Unified Parkinson’s Disease Rating Scale (UPDRS) were recorded. Six patients were classified as neurocognitively normal, six patients as ANI, eight patients as MND, and five patients as HAD. Twenty-two healthy, age and education-matched controls were recruited from the Baltimore, MD, USA community (45–77 years, mean = 60.32 years, 16 females; see Table 1).

**Table 1:** **Healthy controls and Human immunodeficiency virus (HIV+) participants demographic and neuropsychological tests table**.

Demographics, Mean (SD)	Controls (*n* = 22)	HIV+ (*n* = 25)
Males: Females, *n*	6:16	19:06
Age, *years*	60.32 (8.04)	57.96 (7.51)
Education, *years*	15.82 (3.02)	14.58 (2.26)
Duration of HIV infection, *years*	–	20.6 (7.71)
**Neuropsychological tests, Mean (SD)**
CES-depression scale	–	7.68 (9.50)
Digit span test—Forwards	11.00 (2.02)	9.88 (2.64)
Digit span test—Backwards	7.36 (2.36)	6.40 (2.94)
Digit span test—Sum	18.36 (3.86)	16.28 (5.29)
Dominant hand finger tapping	47.12 (8.92)	43.56 (6.72)
Symbol digit modalities test	58.23 (9.81)	54.52 (17.67)

*Controls and HIV+ participants were matched for age and education. None of the variables in the table are significantly different between groups (all *p* values > 0.10)*.

None of the healthy controls reported a history of drug dependance, neurological, mood or psychiatric disorders. All participants were required to pass a urine drug screen on the day of testing (AimScreen MultiDrug 9 by Germaine Laboratories, San Antonio, TX, USA). Additional exclusion criteria for both subject groups were previous or current diagnosis or hospitalization for a major medical condition (besides HIV for the patient group), no history of a severe head injury resulting in a loss of consciousness of more than 5 min or skull fracture. Hepatitis C, a common comorbidity of HIV, was permissible so long as it did not require medication for treatment at the time of testing. This study was approved by the Johns Hopkins Medicine Office of Human Subjects Research Institutional Review Board. Written informed consent was given by all subjects in accordance with the Declaration of Helsinki. Participants were contacted via telephone and email and introduced to the study aims and protocol. If the individual indicated she was interested in the study, a brief telephone screener was administered to determine eligibility for the study. If the volunteer was eligible, she was scheduled for a study appointment. If possible, a consent form was emailed or mailed to the participant prior to the study appointment. On the day of the study visit, the experimenters reviewed the consent form with the volunteer, answered any questions about the study, and allowed for the volunteer to read the entire consent form. Once all questions had been answered to the satisfaction of the volunteer, the experimenter and volunteer signed the consent form together.

### Apparatus

Participants completed all experimental assessments in a well-lit room in the Division of Cognitive Neuroscience located on the medical campus of Johns Hopkins University. The cognitive-motor multitask consisted of drawing on a digitizing tablet and holding information in mind. The task was programmed in MovAlyzeR v6.1 (Neuroscript LLC, Tempe, AZ, USA). Clinical assessments were performed in the Johns Hopkins Institute for Clinical and Translational Research (ICTR) clinic within 6 months of testing.

#### Digitizing Tablet

Digital drawings were recorded using a Wacom Intuos 13″ tablet (48.7 × 31.8 × 1.2 cm). Participants drew on the tablet using a non-marking stylus. All buttons on the stylus were disabled to prevent inadvertent disruptions of task progress. The tablet was connected by USB to a Dell Optiplex 380 where MovAlyzeR was installed and recorded stylus movements along the tablet surface.

#### Kinematic Analysis

Kinematics were partially analyzed using MovAlyzeR software. The sampling rate was set to 100 Hz and a device resolution of 0.0005 cm. MovAlyzeR was programmed to process stylus coordinates with a low-pass filter using a complex fast Fourier transform (FFT) followed by a frequency-domain filter with a filter frequency of 12 Hz and a sinusoidal transition band between 5.1 Hz and 18.9 Hz, followed by the inverse FFT (Teulings and Maarse, 1984). Trailing pen lifts were removed from analyses.

### Cognitive-Motor Multitask Assessment

The current paradigm aimed to examine fine hand and arm movements combined with cognitive demands. This cognitive-motor multitask is distinguished from previous paradigms that have combined cognitive assessments (e.g., Shallice and Burgess, 1991) or emphasized cognitive performance (e.g., Scott et al., 2011). The current cognitive-motor multitask paradigm was defined by three phases: (1) motor-only; (2) cognitive-only; and (3) cognitive-motor combined, with two main conditions: (1) single task (motor or cognitive-only); and (2) multitask (cognitive-motor combined).

#### Motor-Only

**Figure 1:**
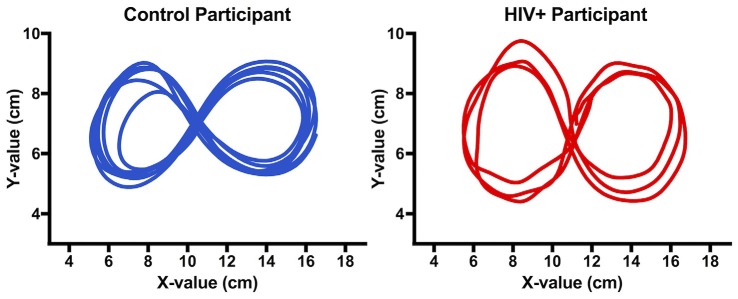
**Figure 8s drawn by a control and human immunodeficiency virus (HIV+) participant during trial 1 of the motor-only phase**. While completing the task, participants did not see the drawings (i.e., it was not displayed on the tablet surface or computer monitor). The images above have been recreated from the raw data of stylus position (cm) on the digitizing tablet.

**Figure 2:**
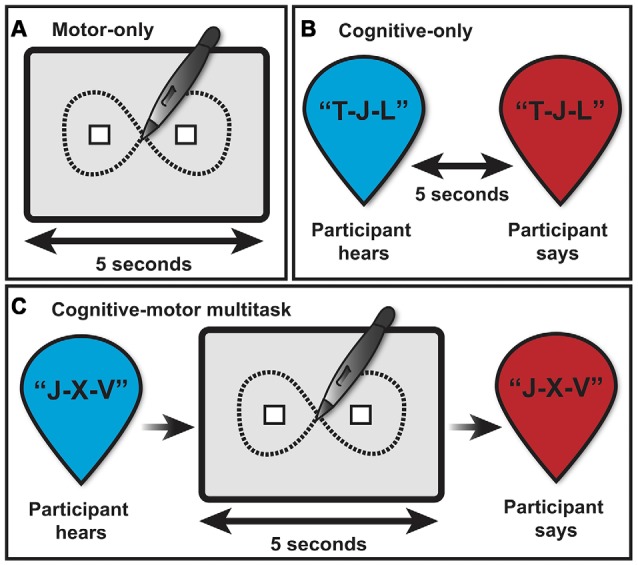
**Depiction of the multitasking assessment consisting of three task phases: (A)** motor-only, **(B)** cognitive-only, and **(C)** cognitive-motor multitask, and two main task conditions: single (motor or cognitive-only) and multitask (cognitive-motor combined).

Participants sat at a desk with the Wacom Intuos digitizing tablet and stylus placed in front of them. Participants were instructed to use the stylus to draw as many continuous (i.e., without lifting the stylus) figure 8s (similar to the infinity symbol: ∞) as possible during a 5-s trial. Examples of these figure 8s drawings are displayed in Figure 1. Accuracy was defined by: (1) drawing within the recording field of the tablet (32.5 × 20.3 cm) indicated by four backlit brackets on the tablet surface; and (2) maintaining general consistency of size and shape of the figure 8s within each trial (e.g., no circles). No specific instructions were given for the dimensions of the figure 8s. To guide the figure 8s and control the minimum figure 8 size, two square stickers (1.27 × 1.27 cm) were affixed to the tablet surface (location: 11.43 × 10.16 cm from either side of the table; see Figure 2). The participants were instructed that the two looped ends of the figure 8 should wrap around the stickers and the intersecting point of the figure 8 should meet at approximately between the stickers. Finally, the volunteers were instructed to avoid contacting the stickers with the stylus while completing the task. There were no penalties or redirections for incorrectly drawing the figure 8s during the test. While the Dell Optiplex 380 was connected to a Dell E171FP monitor positioned in front of the participant, no image or feedback was presented on the monitor during the task. Measures of interest, defined in the “Results” Section, included: (1) the number of figure 8s drawn; (2) velocity of movement (motor speed); and (3) variability, or the coherence among the figure 8s drawn within individual trials.

At the beginning of each trial, participants were instructed to hover 1–2 inches above the tablet surface directly between the stickers. Next, a start command (“start”) cued the subjects to begin drawing the figure 8s directly onto the tablet surface with the stylus. After 5 s of drawing, an end command (a 20 ms 800 Hz tone) indicated the trial was complete, and the participants could remove the stylus from the tablet surface. MovAlyzeR was programmed to begin recording the 5-s trial only when the participant touched the tablet surface with the stylus and automatically stopped recording after 5 s from trial onset, even if the participant continued drawing beyond the stop command. Accordingly, each participant received exactly 5 s per trial, regardless of reaction time to respond to task commands. Each participant completed eight trials of the motor-only phase with approximately 10–20 s breaks between each trial (see Figure 2A).

#### Cognitive-Only

Following the motor-only phase, participants were instructed on a working memory task. The experimenter stated a sequence of letters, administered at 1-s intervals (e.g., T-J-L or K-B-F-T-Q-R-V-N). The letter sequences omitted vowels to prevent participants from generating phonetic non-words as a recall strategy (e.g., F-A-B-E and L-E-K-O). In addition, the number of rhyming letters (e.g., B, C, D, T, V, Z) was controlled by letter sequence length (3-letter sequence = 0 rhymes; 4- and 5-letter sequences = 1 rhyme; 6- and 7-letter sequences = 2 rhymes; 8-letter sequence = 3 rhymes). Letter sequences excluded identical letter orders across sequences (e.g., M-V-*N-S-B* and L-*N-S-B*-W). Thus, unique letter sequences were administered across 12 trials. Sequence length began with a span of three letters and increased by one letter after every two trials, ending with a span of eight letters (see Figure 2B).

After hearing the final letter in a sequence, participants were instructed to silently rehearse the letters (“in your head”) for 5 s. After the 5-s delay, participants were prompted to repeat the letters aloud in the exact same order as presented. Cognitive performance was assessed by: (1) the total number of correctly remembered letters, regardless of order; and (2) the longest sequence of letters given in the correct order, or the maximum number of letters in sequence. Recall scores were averaged across trials of the same letter length.

#### Cognitive-Motor Multitask

The final phase combined the motor- and cognitive-only task. First, subjects heard a novel sequence of letters, as in the cognitive-only phase. After the final letter was administered, a start command cued the participant to begin drawing figure 8s using the stylus and tablet. Identical to the motor-only phase, participants were given 5 s to draw as many continuous figure 8s as possible. Simultaneously, participants silently rehearsed the letter sequence introduced immediately prior. Finally, a stop command prompted the participants to stop drawing and repeat the letter sequence aloud to the experimenter. A total of 12 cognitive-motor trials were completed. As in the cognitive-only phase, the number of letters per sequence ranged between 3–8 letters and increased by one letter every two trials (see Figure 2C).

### Supplemental Assessments

Participants completed several standard assessments of cognitive and motor function. These tests were selected because they are among those commonly used to measure performance in people with HIV to determine a HAND score and predict prognosis (Schouten et al., 2011).

#### Digit Span

Participants completed the Digit Span test from the Wechsler Adult Intelligence Scale-Third Edition (Wechsler, 1997). Participants heard a sequence of numbers and then repeated the sequence aloud either in the same order they were administered (“forward”) or reverse order (“backwards”). There was no delay between the sequence administration and participants’ verbal recall. Total Digit Span scores were calculated by summing forward and backward performances.

#### Finger Tapping

Participants completed the finger tapping task from the Halstead and Reitan Battery (Broshek and Barth, 2000). Participants were instructed to place the palm of their hand on the finger tapping board surface and their index finger on a lever attached to a counter. Subjects were instructed to press the lever as many times as possible during a 10-s interval. Both hands were tested up to 10 trials each. Trials terminated early if the participant maintained the number of lever press performance within 10% for five consecutive trials. The mean number of finger taps per trial were computed for each hand.

#### Symbol Digit Modalities Test (SDMT)

The primary neurocognitive function assessed with Symbol Digit Modalities Test (SDMT) is processing speed, although working memory and attention are involved in the task (Smith, 1982). Participants are shown a key that matches symbols with the numbers 1–9 and a set of symbols missing their corresponding number. Participants were instructed to use the key to identify the appropriate number for each symbol and state their responses, which were recorded by the experimenter. Thus motor involvement was minimized. Scores on the SDMT were based on the total number of correct responses within a 2-min time limit.

#### The Center for Epidemiologic Studies Depression Scale (CES-D)

Depression is the most common neuropsychiatric disorder among people with HIV, impacting approximately half of all patients (Almeida, 2013; Nanni et al., 2015). It can be challenging to differentiate the contribution of HIV from depression in driving neurocognitive impairments (Nanni et al., 2015). To control for depression, all HIV+ participants were administered the Center for Epidemiologic Studies Depression Scale (CES-D) that gauges symptoms of depression through a 20-item self-report scale (e.g., “I felt lonely” and “I talked less than usual”; Radloff, 1977). Scores less than 16 indicate no depression symptomatology. Healthy controls were not administered the CES-D as they were excluded for a diagnosis of mood or psychiatric disorders.

### Procedures

After consenting and drug screening, participants performed the cognitive-motor multitask. Next, participants completed the supplemental assessments of working memory (Digit Span test), motor performance (finger tapping and SDMT) and mood (CES-D, patients only). All data were collected within one test session.

### Data Analysis

**Figure 3:**
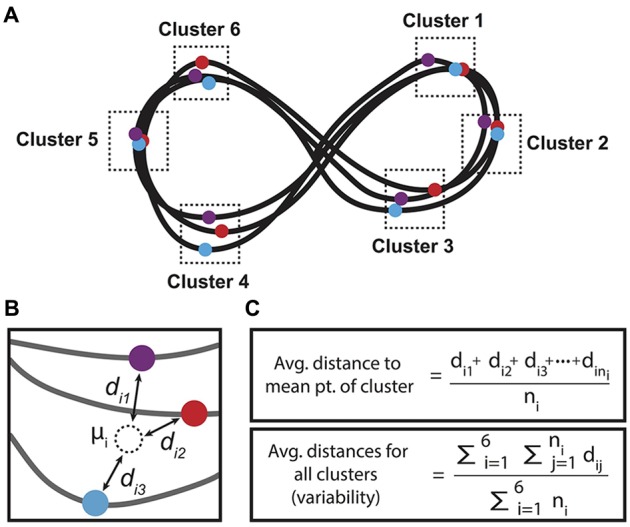
**Method for calculating figure 8 variability, or drawing quality. (A)** Figure 8s were divided into six clusters where the *x*- or *y*-coordinate met a maximum or minimum value for each individual figure 8 drawn per trial. **(B)** Within each cluster, a mean point was calculated, and the average distance of the cluster’s extreme points to the mean point was calculated. **(C)** Formula used for calculating the average distance to the mean point of each cluster and the average distances for all clusters, or the variability value per trial. Note that n_i_ is the number of points in the i-th cluster.

The motor performance was measured by the total number, velocity and variability of figure 8s drawn within each trial. The number of figure 8s drawn were counted by reviewing all drawings per trial, dividing the figure 8s into eight segments defined by the regions between the maximum and minimum x- and y-values and the central point (four per looped end of the figure 8) and counting the number of fully completed segments. The velocity values were generated by MovAlyzeR using the function $\surd [\text{x(cm}/{\text{s)}}^{\text{2}}+\text{y(cm}/{\text{s)}}^{\text{2}}]$. Variability evaluated the relative level of heterogeneity among figure 8s drawn within a single trial and represents a measure of drawing quality. Variability was calculated by querying all peak and nadir *x*- and *y-coordinates* for each individual figure 8 drawn (Figure 3A). These coordinates were defined within six regions of interest within the figure 8. A mean coordinate for the peak or nadir points was calculated per region, from which the distance (cm) between the mean coordinate and all the points that defined that mean were found (Figure 3B). The distances were averaged across all six figure 8 regions per trial resulting in a single value for figure 8 variability for each trial, per subject (Figure 3C). On rare occasions when only one point was available within a region of interest (i.e., the number of figure 8s drawn was <2) this region was omitted from variability analysis. In order to include a HIV+ participant with a trial recording error (multitask trial 4) in mixed design analyses (ANOVA), mean performance from all other multitask trials replaced the omitted value.

## Results

### Cognitive-Motor Multitask

#### Motor Performance

**Figure 4:**
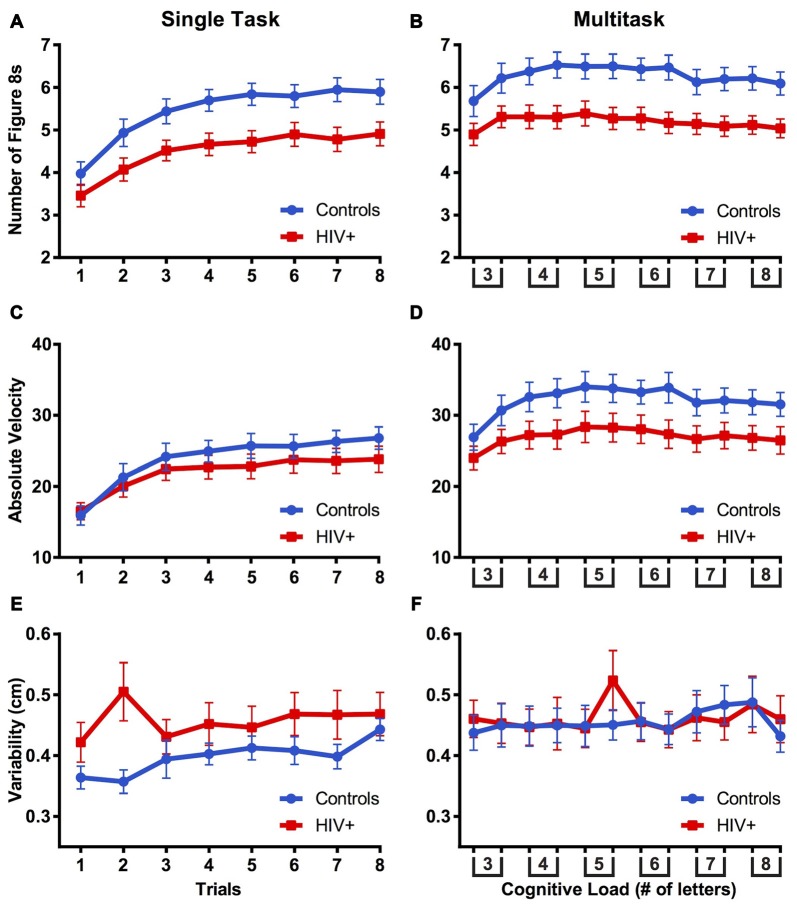
**The mean number of figure 8s drawn (A,B)**, mean velocity **(C,D)**, and mean variability **(E,F)** between controls and HIV+ participants compared across single and multitasks phases. Error bars represent standard error of the mean.

The number of figure 8s drawn were evaluated using a mixed-design ANOVA with group (control vs. HIV+) as a between-subjects factor and trial number and condition (single vs. multitask) as within-subjects factors. Due to the necessity for an equal number of trials between conditions, analyses were completed with trials 1–8 of single task and trials 1–8 of multitask. Mauchly’s Test of Sphericity indicated that the assumption of sphericity was violated for trial, $\chi_{\text{(27)}}^{\text{2}}$ = 168.96, *p* < 0.001, and condition by trial, $\chi_{\text{(27)}}^{\text{2}}$ = 146.00, *p* < 0.001, therefore Greenhouse-Geisser estimates of sphericity (*ε* = 0.355, *ε* = 0.411, respectively) were used to correct for degrees of freedom. As can be seen in Figures 4A,B, there was a main effect of trial, *F*_(2.48,111.69)_ = 58.05, *p* < 0.001, $\eta_{\text{p}}^{\text{2}}$ = 0.563, confirming that the number of figure 8s drawn increased with practice. There was a main effect of condition, *F*_(1,45)_ = 47.21, *p* < 0.001, $\eta_{\text{p}}^{\text{2}}$ = 0.512, indicating that the number of figure 8s drawn was greater in the multitask than single task condition. A main effect of groups, *F*_(1,45)_ = 8.19, *p* = 0.006, $\eta_{\text{p}}^{\text{2}}$ = 0.154, revealed that HIV+ participants drew fewer figure 8s than did controls. There was an interaction of condition by trial, *F*_(2.88,129.50)_ = 15.74, *p* < 0.001, $\eta_{\text{p}}^{\text{2}}$ = 0.259, indicating that the number of figures 8s drawn increased more quickly across trials of the single task condition than in the multitask condition. Finally, there was an interaction of group by trial, *F*_(2.48,111.69)_ = 2.88, *p* = 0.049, $\eta_{\text{p}}^{\text{2}}$ = 0.060, revealing that figure 8 counts increased more sharply for controls than for patients across task trials.

Velocity data were analyzed using a mixed-design ANOVA with group (control vs. HIV+) as a between-subjects factor and trial (single task: trials 1–8; multitask: trials 1–8) and condition (single vs. multitask) as within-subjects factors. In these analyses, the assumption of sphericity was violated, trial, $\chi_{\text{(27)}}^{\text{2}}$ = 212.11, *p* < 0.001, and condition by trial, $\chi_{\text{(27)}}^{\text{2}}$ = 131.05, *p* < 0.001, therefore degrees of freedom were corrected using Greenhouse-Geisser estimates of sphericity (*ε* = 0.340, *ε* = 0.430, respectively). A main effect of trial, *F*_(2.38,107.17)_ = 57.42, *p* < 0.001, $\eta_{\text{p}}^{\text{2}}$ = 0.561, showed that the velocity increased with practice. A main effect of condition, *F*_(1,45)_ = 68.12, *p* < 0.001, $\eta_{\text{p}}^{\text{2}}$ = 0.602, indicated that the velocity was greatest during the multitask condition. There were significant interactions of condition by trial, *F*_(3.01,135.51)_ = 4.77, *p* = 0.003, $\eta_{\text{p}}^{\text{2}}$ = 0.096, group by trial, *F*_(2.38,107.17)_ = 2.91, *p* = 0.050, $\eta_{\text{p}}^{\text{2}}$ = 0.061, and trending group by condition, *F*_(1,45)_ = 3.98, *p* = 0.052, $\eta_{\text{p}}^{\text{2}}$ = 0.081. These interactions reflect that velocity increased in the single task more than multitask condition but controls showed greater velocity increases across trials and between task conditions compared to the HIV+ participants (Figures 4C,D). These results supported the analysis of mean velocity (mean [standard deviation]) revealing no difference between controls and patients in the single task (23.84 [7.08] and 21.95 [7.62] cm/s, respectively), yet a trending difference between controls and patients in the multitask (32.12 [8.79] and 26.99 [9.33] cm/s, respectively), *t*_(45)_ = 1.93, *p* = 0.060. This further supports that controls were faster than patients while multitasking.

Variability (figure 8 quality) was analyzed using a mixed-design ANOVA with group (control vs. HIV+) as a between-subjects factor and multitask trial (single task: trials 1–8; multitask: trials 1–8) and condition (single vs. multitask) as within-subjects factors. The assumption of sphericity was violated, condition by trial, $\chi_{\text{(27)}}^{\text{2}}$ = 60.07, *p* < 0.001, therefore degrees of freedom were corrected using Greenhouse-Geisser estimates of sphericity (*ε* = 0.728). A trending main effect of condition, *F*_(1,45)_ = 3.86, *p* = 0.056, $\eta_{\text{p}}^{\text{2}}$ = 0.079, suggested that variability was greater during multitask compared to single task. There was a trending group by condition interaction, *F*_(1,45)_ = 3.205, *p* = 0.080, $\eta_{\text{p}}^{\text{2}}$ = 0.066, suggesting that variability was less for controls in the single task than in the multitask condition (Figures 4E,F).

**Figure 5:**
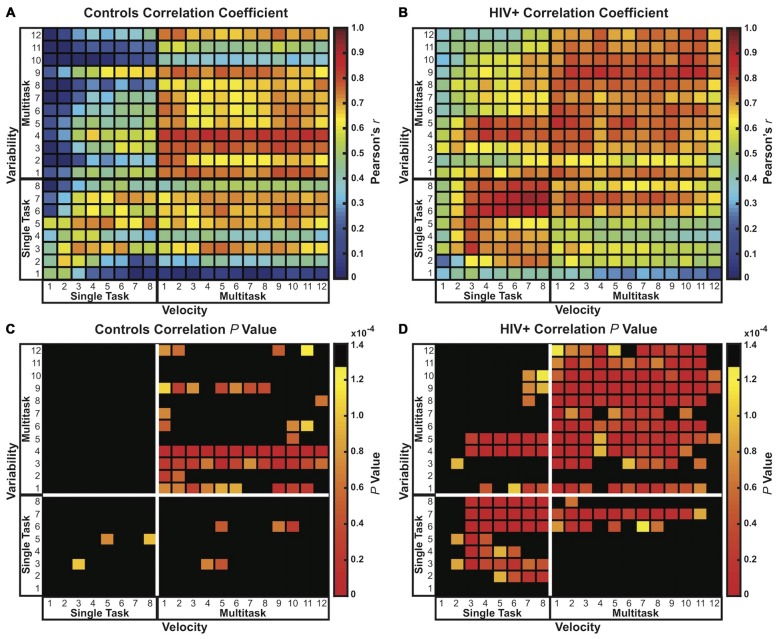
**Correlation coefficient matrices (20 × 20) comparing velocity and variability**. Pearson’s *r* and *p* value matrices are plotted for controls **(A,C)** and HIV+ participants **(B,D)**, respectively. Both groups showed positive correlations between velocity and variability, with strongest Pearson’s *r* and *p* values shown in red. HIV+ participants demonstrated a notably strong positive link between velocity and variability compared to controls. The critical *p* value was adjusted using the Bonferroni correction for multiple comparisons resulting in a corrected threshold of *p* < 0.000125 (0.05/400).

Pearson’s bivariate correlations analysis of velocity and variability across *all* trials of single and multitask conditions (20 × 20 correlation matrix) revealed a positive relationship between velocity and variability for both participant groups (Figures 5A,B). As velocity increased, so did the variability of the figure 8s. However, Figure 5 also demonstrates that the link between variability and velocity is stronger for HIV+ participants than for controls, with 15.00% of correlations (60/400) for controls and 47.75% of correlations (191/400) for patients resulting in a statistically significant interaction, surviving a Bonferroni correction for multiple comparisons, *p* values < 0.000125 (0.05/400) (Figures 5C,D). In other words, motor performance by controls on any one trial was less predictive of performance on subsequent or preceding trials than it was for patients. Moreover, single task velocity was more predictive of multitask variability in patients than in controls. Taken together these results indicated that controls were better able to maintain low variability while increasing speed compared to patients. Thus, for the HIV+ participants, increasing speed with practice across trials came at a cost of figure 8 drawing quality.

#### Cognitive Performance

The total number of letters recalled in working memory was analyzed using a mixed-design ANOVA with group (control vs. HIV+) as a between-subjects factor and trial (3, 4, 5, 6, 7 and 8 letter trials) and condition (single vs. multitask) as within-subjects factors. The assumption of sphericity was violated for trial,$\chi_{\text{(27)}}^{\text{2}}$ = 48.48, *p* < 0.001, and condition by trial, $\chi_{\text{(27)}}^{\text{2}}$ = 28.98, *p* = 0.011, therefore Greenhouse-Geisser estimates of sphericity (*ε* = 0.678, *ε* = 0.839, respectively) was used to correct for degrees of freedom. Main effects of condition, *F*_(1,45)_ = 30.98, *p* < 0.001, $\eta_{\text{p}}^{\text{2}}$ = 0.408, and trial, *F*_(3.39,152.55)_ = 123.22, *p* < 0.001, $\eta_{\text{p}}^{\text{2}}$ = 0.732, were associated with an interaction of condition by trial, *F*_(4.19,188.72)_ = 6.35, *p* = 0.001, $\eta_{\text{p}}^{\text{2}}$ = 0.124, indicating that letter recall declined more quickly across trials in the multitask than single task condition.

**Figure 6:**
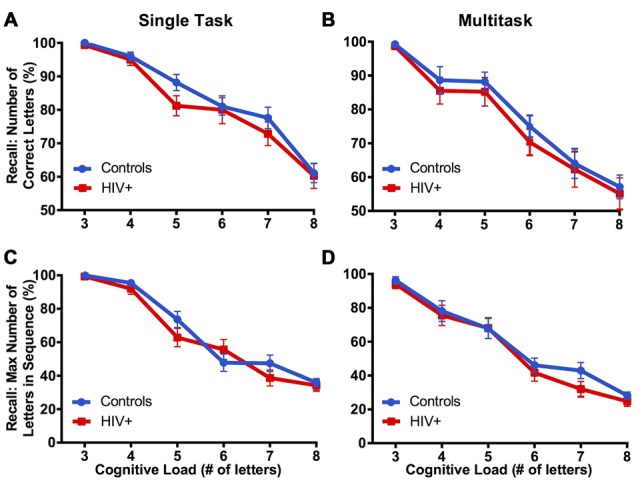
**Cognitive performance of controls and HIV+ participants during cognitive-only (A,C)** and cognitive-motor combined **(B,D)** phases of the multitask paradigm, standard error of the mean is reported. Groups did not differ in cognitive performance during single or multitask conditions, as measured by total number of letters recalled and maximum number of letters recalled in sequence.

The maximum number of letters recalled in sequence was analyzed using a mixed-design ANOVA with group (control vs. HIV+) as a between-subjects factor and task trial (3, 4, 5, 6, 7 and 8 letter trials) and condition (single vs. multitask) as within-subjects factors. The findings were repeated from the total number of letters recalled described above, all *p* values < 0.05. Groups did not differ on both measures of recall performance across conditions or trials (Figure 6).

**Figure 7:**
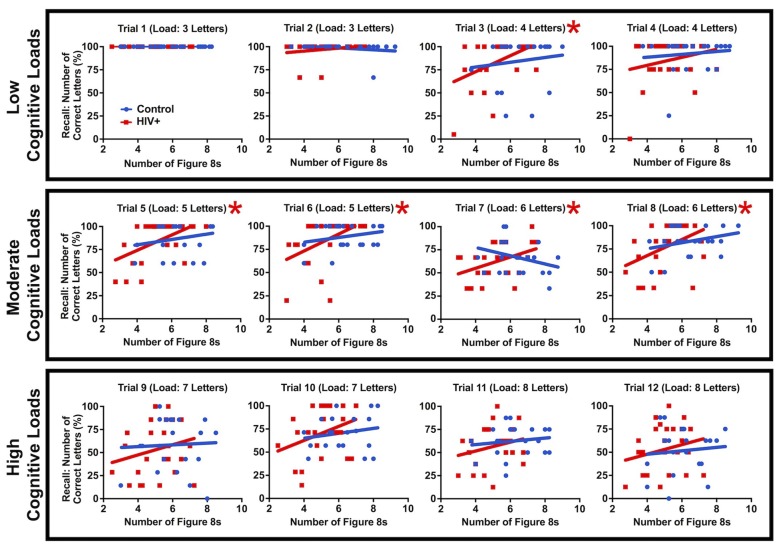
**The number of figure 8s drawn vs. letter recall by individual for all trials of the multitask condition**. A fitted linear regression line is plotted for each group and a red asterisk indicates that the patient regression line is significantly different from zero (*p* < 0.05). Patients reveal a positive relationship between recall and figure 8s drawn for moderate cognitive loads driven by notably poor HIV+ multitaskers. Controls do not show this relationship (*p* > 0.10).

**Figure 8:**
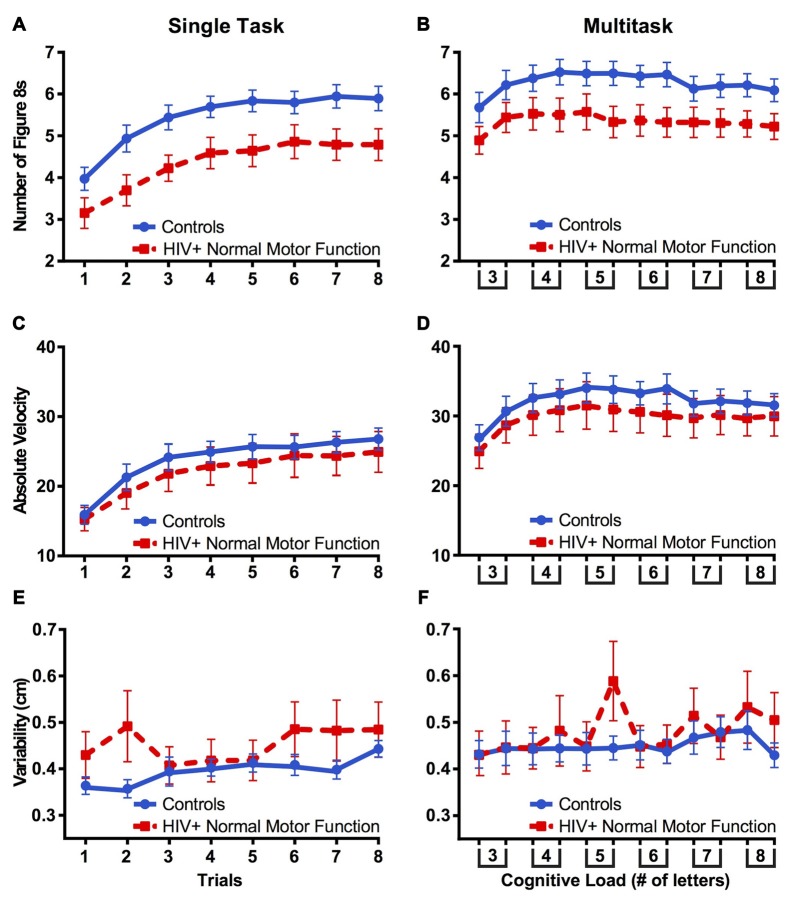
**Fourteen HIV+ participants classified with normal motor function via the Unified Parkinson’s Disease Rating Scale (UPDRS) and supplementary motor function exams (red dashed line) drew fewer figure 8s compared to healthy controls (A,B)**. Groups did not differ in terms of absolute velocity **(C,D)** or variability **(E,F)**. These results indicate that a “normal” neurological exam of motor function may not capture subtle motor impairments when performing fine motor tasks or a motor task combined with a cognitive load. Error bars represent standard error of the mean.

Although group cognitive performance was statistically identical between patients and controls, differences were found in the relationship between working memory and motor performance. A linear regression was calculated between letter recall and number of figure 8s drawn while multitasking and patients revealed a positive interaction for moderate cognitive loads, trial 3, *F*_(1,23)_ = 5.96, *p* = 0.023, trial 5, *F*_(1,23)_ = 9.34, *p* = 0.0045, trial 6, *F*_(1,23)_ = 6.49, *p* = 0.018, trial 7, *F*_(1,23)_ = 5.001, *p* = 0.035 and trial 8, *F*_(1,23)_ = 5.069, *p* = 0.034 (Figure 7). This relationship was not found for controls (linear regression slopes fitted to the data were not significantly different from zero, *p* values > 0.10). These results were driven by a subset of patients with both poor motor and working memory performance. At low and high cognitive loads, working memory performance was either at ceiling or compromised across all patients, respectively, eliminating the positive interaction. This result supports a link between cognitive and motor impairment in HIV and that a subset of HIV+ participants were particularly challenged by cognitive-motor multitasking.

### Supplemental Tests

Independent-samples *t*-tests showed that groups did not differ on measures of hand agility (finger tapping task), processing speed (SDMT), or working memory capacity (Digit Span) (Table 1). CES-D did not correlate with any cognitive measure of the multitask paradigm for patients, all *p* values > 0.05, and sparsely correlated with motor measures (trial 6 multitask for the number of figure 8s drawn, trial 2 single task variability, and trials 1, 5 and 10 multitask variability, *p* values < 0.05), otherwise all *p* values > 0.05. This demonstrates that depression symptomology had minimal impact on motor and cognitive performance.

#### HAND Correlates of Cognitive-Motor Multitask and Supplemental Test Performance

HAND scores did not correlate with the number of figure 8s drawn, velocity, or variability performances on single or multitask conditions, *p* > 0.05. For cognitive measures, HAND scores did not correlate with the number of letters and maximum number of letters recalled in sequence for either the single or multitask conditions, *p* values > 0.05, with the exception of 3-letter multitask trials for maximum letter sequence, *p* = 0.041. HAND score negatively correlated with SDMT (higher HAND was associated with slower processing speed), *r*_(25)_ = −0.537, *p* = 0.006, and positively correlated with CES-D (higher HAND scores were linked with greater symptoms of depression), *r*_(25)_ = 0.533, *p* = 0.006. HAND did not predict performance on other supplemental tests (finger tapping and Digit Span), all *p* values > 0.05.

#### Validity of Neuro-Medical Exams of Motor Function in HIV

All patients were evaluated for motor function through the Johns Hopkins HIV Neurology Clinical Core Cohort. Assessments included the UPDRS and supplementary ratings of coordination, gait, and tremor. Fourteen HIV+ participants were classified as normal on both the UPDRS and supplementary motor function exams. Patients with normal motor function were compared with controls for number of figure 8s drawn using a mixed-design ANOVA with group (control vs. motor normal HIV+ participants) as a between-subjects factor and trial (single task: trials 1–8 vs. multitask: trials 1–8) and condition (single vs. multitask) as within-subjects factors. The assumption of sphericity was violated for trial, $\chi_{\text{(27)}}^{\text{2}}$ = 137.28, *p* < 0.001, and condition by trial, $\chi_{\text{(27)}}^{\text{2}}$ = 126.03, *p* < 0.001, therefore degrees of freedom were corrected using Greenhouse-Geisser estimates of sphericity (*ε* = 0.344, *ε* = 0.368, respectively). A group effect was found, *F*_(1,34)_ = 5.85, *p* = 0.021, $\eta_{\text{p}}^{\text{2}}$ = 0.147, in the direction of HIV+ patients drawing fewer figure 8s than did controls (Figures 8A,B).

To compare figure 8 variability between controls and clinically motor normal HIV+ participants, a mixed-design ANOVA was utilized with group as a between-subjects factor and trial (single task: trials 1–8 vs. multitask: trials 1–8) and condition (single vs. multitask) as within-subjects factors. The assumption of sphericity was violated for condition by trial, $\chi_{\text{(27)}}^{\text{2}}$ = 62.41, *p* < 0.001, therefore degrees of freedom were corrected using Greenhouse-Geisser estimates of sphericity (*ε* = 0.669). While there was no group effect for variability, there was a significant group by trial interaction *F*_(7,238)_ = 2.30, *p* = 0.028, $\eta_{\text{p}}^{\text{2}}$ = 0.063, indicating that variability for controls increased across trials more than for patients (Figures 8E,F). Velocity measures did not differ between groups (Figures 8C,D), all *p* values > 0.05.

## Discussion

This study demonstrated that HIV+ participants were impaired on motor performance while multitasking. The patient and control groups differed by the number of figure 8s drawn for both the motor-only and multitask conditions. This was evident even in the subgroup of HIV+ individuals who showed no signs of motor impairments in their clinical assessments. These results replicate previous research that found deficits of fine motor movements among HIV+ individuals who were asymptomatic on clinical tests of neuropsychological function (Arendt et al., 1990). Our data present the possibility that motor impairment in the multitask could have been driven by baseline motor impairments of the HIV+ participants, or as the result of combining motor and cognitive tasks. These results support the latter by showing that motor speed (velocity) was only impaired among patients during the multitask condition, indicating that multitasking was a primary driver of their diminished motor performance. Previous accounts of HIV+ participants with multitasking deficits (e.g., Schouten et al., 2011) support these current findings.

While the HIV+ participants were impaired overall in motor speed, both controls and patients revealed similar patterns of velocity change in their cognitive-motor multitask performance. The increase in motor speed during the multitask condition may represent participants automating motor behavior to release attention and recruit cognitive networks to focus on the working memory task (Dux et al., 2009). However, controls increased velocity in the multitask more than the HIV+ group did, suggesting that HIV+ participants struggled to automate the motor task. As a result, they were less successful in executing the motor portion of the multitasking paradigm. This may explain challenges HIV+ patients report in their daily and professional lives that require attentional switching and prioritization that is common with multitasking (Schouten et al., 2011).

We expected patients to show less variability than controls in association with the lower velocity in the HIV+ group while multitasking. However, each group performed the motor task with equal variability during single and multitask conditions. Moreover, velocity more strongly predicted variability in patients than controls, indicating that patients were less able to maintain fine motor performance as speed increased, particularly while multitasking. Meanwhile, less coupled velocity and variability performance in controls compared to patients revealed controls increased motor speed with less of an impact on motor quality. These results suggest that when faced with a multitask challenge in daily life, an individual with HIV is more likely to slow down to control for errors.

In addition, our findings demonstrate that standardized assessments of motor impairments in HIV may lack sensitivity. First, finger tapping, a common tool to access motor function, did not differentiate between groups. Moreover, of the HIV+ participants recruited for this study, more than half were determined to have normal motor function according to clinic evaluations. However, performance by these same patients on the multitask paradigm revealed fewer figure 8s drawn in single and multitask conditions relative to controls. These results show that common assessments of motor function in HIV can be improved by: (1) utilizing a fine motor task; and (2) pairing motor assessments with a competing or distractor task that helps to minimize or tax the use of compensatory mechanisms that otherwise aid patients to maintain “normal” performances in the clinic.

Contrary to our hypothesis, patient working memory performance did not differ from that of controls. Increased cognitive load and multitasking reduced recall but equally between groups. This outcome is inconsistent with previous studies showing working memory deficits in HIV (Sundermann et al., 2015). There are several possible explanations for these results, including: (1) the working memory task was not sensitive enough to differentiate between controls and patients; (2) the patients may have prioritized cognitive over motor performance thereby holding recall on par with controls while sacrificing motor performance; or (3) individual-based differences may exist that distinguish a subset of HIV+ participants but are masked at group-level analyses. The latter explanation is supported by a patient-specific positive interaction between motor and working memory performance while multitasking at intermediate cognitive loads. This interaction was driven by HIV+ participants with exceptional multitasking deficits. These results support previous work revealing cognitive, motor and multitasking deficits in HIV. Additional investigations are necessary to determine if the multitasking impairments shown here relate to clinical variables and are predictive of function in daily life. Moreover, manipulations of the cognitive-motor task (e.g., changing the cognitive task, or explicitly instructing participants on task priorities) may help to emphasize the cognitive deficits in HIV and more reliably identify those patients with neurocognitive impairments.

The AAN and updated Frascati criteria for the neurocognitive stages of HIV share a reliance on standardized testing of cognitive and motor functions (Janssen et al., 1991; Antinori et al., 2007). This is reflected by the criteria that define impairments through domain-specific deficits. These standards neglect patients who are normal on clinical assessments of cognition and motor function but fail to maintain performance when cognitive and motor loads are required simultaneously. The addition of clinical multitasking tests and corresponding criteria to define HIV-associated multitasking dysfunctions may allow for earlier detection of subtle neurocognitive impairments or patients whose deficits are most prevalent at the intersection of cognitive-motor demands. Likewise, multitasks may offer increased sensitivity among the HAND stages, perhaps expanding the current four-point scale to include intermediate neurocognitive impairment transitions. For example, a pre-ANI phase may be defined among the HIV+ patients who are normal on in clinic assessments but impaired on multitasks, potentially predicting that these individuals are at risk to transition to ANI in the future. The clinical utility of multitasks in HIV is also supported by a previous report showing that performance on a multitask better predicted impairments in daily life than did single modality neurocognitive tests (Scott et al., 2011).

While the data from the current investigation does not directly reflect underlying mechanisms for HAND, the results would be explained if HIV targeted neural networks that support multitasking, particularly those that incorporate both cognitive and motor functions. For example, damage to a cognitive-motor network may result in sustained “normal” motor and cognitive performance under single task conditions but show deficits while multitasking due to greater network demands. A prominent dual cognitive and motor structure is the cerebellum. The cerebellum is established as foundational to motor function and more recently shown to support a variety of cognitive functions, including emotional regulation, executive functions, language and memory (Koziol et al., 2014). In addition, the cerebellum is active while multitasking for both cognitive-cognitive (Deprez et al., 2013) and cognitive-motor multitasks (Wu et al., 2013). Indeed, damage or degeneration of the cerebellum can result in challenges with multitasking, for example as seen in patients with spinocerebellar ataxia (Brusse et al., 2011).

While HIV is commonly not considered a disorder of the cerebellum, previous investigations show that the cerebellum is injured by HIV. A neuroimaging study found correlations between motor deficits in HIV and pontocerebellar tissue volume loss (Sullivan et al., 2011). Moreover, an investigation of the simian immunodeficiency virus (SIV) found significant granule cell loss from early stages of infection and Purkinje cell density reduction at late stages of the SIV disease process (Wáchter et al., 2016). Likewise, degeneration of the cerebellum and granule cell loss are found in patients with HIV (Tagliati et al., 1998; Sclar et al., 2000). Alongside cerebellar impairments, the basal ganglia is targeted by HIV and also contributes to multitasking (Thoma et al., 2008). Therefore, multitask deficits in HIV may be explained by both cerebellar and basal ganglia network damages. Additional investigation of tissue volume and functional activation profiles of the cerebellum and basal ganglia in relation to multitasking performance in HIV will be crucial to establish these links.

### Study Limitations

A primary limitation of this investigation is non-matched gender proportions among controls and patients. The Johns Hopkins HIV Neurology Clinical Core Cohort from which the HIV+ participants were recruited were predominantly homosexual males. While modern trends in the United States show increased HIV transmission attributed to heterosexual contact, which accounts for 74% of infections among women, the majority of newly infected HIV patients continue to occur through male-to-male sexual contact (Centers for Disease Control and Prevention, 2014). These trends are represented in the current study as the majority of HIV+ participants are males, yet approximately a third of controls are male. While this presents a potential confound between groups, statistical analyses revealed that the current results were not driven by sex differences. The role of sex differences in HIV/AIDS is uncertain, with some investigations revealing group differences in neurocognitive performance (Hestad et al., 2012), while others finding no evidence for sex influencing the progress of the disease (Robertson et al., 2004).

## Conclusion

This investigation aimed to evaluate motor and cognitive function in HIV using a novel paradigm that is more ecologically valid than most standard neurocognitive tests. The results demonstrated motor deficits using sensitive quantitative measures, especially when combined with a cognitive load, in HIV+ patients compared to controls. The motor impairments were not predicted by clinical evaluations of motor function. This suggests that common assessments of motor function in HIV used in outpatient settings may not be sensitive to early stages of disease progression. Adopting similar task parameters as in the current study could improve early detection of motor impairments and refine prognosis. Future investigations should aim to better understand the relationship between cognitive and motor impairments in HAND, their underlying mechanisms (e.g., damage to the cerebellum and basal ganglia), and the predictive value of multitasks when assessing current disease state and future outcomes.

## Author Contributions

SIK, NCS and CLM designed the study, while SIK and CLM created the methodology and collected the data. SIK, JAM and CLM performed the analyses. SIK, CLM and NCS interpreted the results. SIK and CLM drafted the manuscript. SIK, JAM, NCS and CLM edited and approved the final version of the manuscript.

## Funding

This research was supported by Office of Extramural Research, National Institutes of Health (NIH; K01 DA030442, P30 MH075673) and the Margaret Q. Landenberger Research Foundation. Additionally, this publication was made possible by the Johns Hopkins Institute for Clinical and Translational Research (ICTR) which is funded in part by Grant Number UL1 TR 001079 from the National Center for Advancing Translational Sciences (NCATS) a component of the National Institutes of Health (NIH), and NIH Roadmap for Medical Research. Its contents are solely the responsibility of the authors and do not necessarily represent the official view of the Johns Hopkins ICTR, NCATS or NIH.

## Conflict of Interest Statement

The authors declare that the research was conducted in the absence of any commercial or financial relationships that could be construed as a potential conflict of interest.
